# Herbal Extracts and Plant‐Derived Bioactives in Atopic Dermatitis: Evidence, Mechanisms, Pediatric Safety, and Translational Priorities

**DOI:** 10.1002/fsn3.72239

**Published:** 2026-09-11

**Authors:** Muhuang Zeng, Qianlan Yuan, Shuojia Huang, Xiaofei Lu

**Affiliations:** ^1^ Shantou Hospital of Traditional Chinese Medicine Shantou China; ^2^ Sichuan Jinxin Xinan Women and Children Hospital Chengdu China; ^3^ Guangdong Chaozhou Health Vocational College Chaozhou China; ^4^ Shenzhen Bao'an Chinese Medicine Hospital The Seventh Clinical College of Guangzhou University of Chinese Medicine Shenzhen China

**Keywords:** atopic dermatitis, dietary bioactives, drug delivery, functional foods, gut‐skin axis, herbal medicine, nutraceuticals, pediatric safety

## Abstract

Atopic dermatitis (AD) is a chronic, relapsing epithelial and neuroimmune disease in which barrier failure, type 2 inflammation, microbial dysbiosis, itching, metabolism, and environmental exposures reinforce one another. This review critically synthesizes clinical and preclinical evidence for herbal medicines, standardized extracts, food‐derived bioactives, functional‐food candidates, nutraceutical ingredients, and natural‐product delivery systems in AD, with particular attention to pediatric safety. Recurrent experimental signals include attenuation of Th2 cytokines and IgE, suppression of mast‐cell and IL‐31‐related itch pathways, restoration of filaggrin and tight‐junction proteins, and modulation of MAPK/NF‐κB, JAK/STAT, Nrf2/HO‐1, and NLRP3 signaling. However, efficacy is not inferred from pathway changes alone. Human evidence remains heterogeneous, and apparently promising agents have failed to outperform active comparators, produced neutral microbiome results, or raised barrier and sensitization concerns. We therefore integrate four issues that are often treated separately: food sources and processing‐dependent chemistry; oral bioaccessibility and food‐matrix effects; gut‐skin signaling and its uncertain causal direction; and compatibility with standard topical agents, cyclosporine, biologics, and JAK inhibitors. The evidence supports selected botanicals as investigational adjuncts rather than as replacements for guideline‐based care. Translation requires authenticated materials, quantitative chemical fingerprints, exposure‐response studies, age‐appropriate safety surveillance, interaction assessment, and controlled trials using validated clinical and barrier endpoints.

## Graphical abstract

Food‐derived bioactives, herbal extracts, and standardized botanical products are being investigated as adjunctive strategies for atopic dermatitis. Their biological activity is shaped not only by plant source and nominal dose, but also by fermentation, drying, heating, extraction, food‐matrix binding, gastrointestinal transformation, and formulation. Candidate products may act across barrier, immune, itch, and microbiota‐related axes; nevertheless, clinical benefit depends on reproducible exposure, validated endpoints, and compatibility with established therapy.

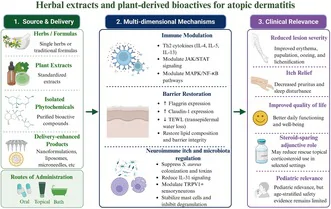

**Abbreviations**
AD: atopic dermatitis
AP‐1: activator protein 1
CYP: cytochrome P450
DNCB: 2,4‐dinitrochlorobenzene
DNFB: 2,4‐dinitrofluorobenzene
EASI: Eczema Area and Severity Index
FMT: fecal microbiota transplantation
HDM: house dust mite
HO‐1: heme oxygenase 1
HPLC: high‐performance liquid chromatography
IFN: interferon
IgE: immunoglobulin E
IL: interleukin
JAK: Janus kinase
KLK7: kallikrein‐related peptidase 7
LC–MS: liquid chromatography–mass spectrometry
MAPK: mitogen‐activated protein kinase
MC903: calcipotriol
MRGPRX2: Mas‐related G‐protein‐coupled receptor X2
NF‐κB: nuclear factor kappa B
NLRP3: NOD‐like receptor family pyrin domain‐containing 3
Nrf2: nuclear factor erythroid 2‐related factor 2
NRS: numerical rating scale
ORAI: calcium release‐activated calcium channel protein
PAR2: protease‐activated receptor 2
P‐gp: P‐glycoprotein
RCT: randomized controlled trial
SCORAD: SCORing Atopic Dermatitis
SIRT1: sirtuin 1
SPINK5: serine peptidase inhibitor Kazal type 5
STAT: signal transducer and activator of transcription
TCM: traditional Chinese medicine
TEWL: transepidermal water loss
Th: T helper
TIS: Three‐Item Severity score
TNF: tumor necrosis factor
TRP: transient receptor potential
TRPM8: transient receptor potential melastatin 8
TRPV1: transient receptor potential vanilloid 1
TSLP: thymic stromal lymphopoietin
vIGA‐AD: validated Investigator Global Assessment for Atopic Dermatitis

## 1 Introduction

Atopic dermatitis (AD) is a chronic relapsing inflammatory skin disease characterized by xerosis, eczematous lesions, severe pruritus, sleep impairment, and recurrent flares. Guideline‐based management prioritizes barrier repair, topical anti‐inflammatory therapy, trigger control, and proactive relapse prevention, with phototherapy, biologics, or Janus kinase (JAK) inhibitors used when disease remains uncontrolled (Chu et al. 2024; Sidbury et al. 2023; Davis et al. 2024). Within food science, a relevant but underdeveloped question is whether defined edible plants, food‐processing by‐products, and dietary bioactives can be developed as functional foods, nutraceuticals, or standardized topical ingredients that support, rather than replace, this therapeutic hierarchy. In this review, “functional food” denotes a consumed food or food‐derived preparation investigated for health effects beyond basic nutrition; “nutraceutical” denotes a concentrated food‐derived ingredient presented in a medicinal format; and “dietary bioactive” denotes a nonessential food constituent with measurable biological activity. These categories overlap with herbal medicines but are not interchangeable, because edible source, processing history, food matrix, route of administration, and regulatory status determine exposure and safety. The network biology of AD makes polyphenols, terpenoids, saponins, glycosides, fatty acids, and polysaccharides scientifically relevant, yet pleiotropic in vitro activity cannot substitute for product definition or clinical proof (Lee, Kim, et al. 2024; Amatto et al. 2024; Jia et al. 2023; Nie et al. 2025; Bai et al. 2025).

The pediatric context deserves separate emphasis. Children are not small adults with eczema. They have age‐specific barrier maturation, body‐surface‐to‐weight ratios, exposure patterns, caregiver‐administered products, higher vulnerability to topical irritants, and frequent overlap with food allergy, asthma, or allergic rhinitis. Complementary and alternative medicine use in pediatric AD is common, but parents often use botanical or food‐derived topical agents without formal dermatologic supervision (Adler‐Neal et al. 2019; Hon et al. 2022; Chen et al. 2016). This creates a dual obligation: to avoid dismissing potentially useful low‐cost adjuncts, and to avoid normalizing unstandardized or allergenic home preparations. In infants and children, an intervention should clear a higher threshold for product identity, contaminant testing, cutaneous tolerance, and adverse‐event capture than in exploratory adult studies (Guo et al. 2022; Karagounis et al. 2019; Bhanot et al. 2019).

This review uses a two‐axis framework. The first axis separates whole formulae, defined extracts, purified compounds, edible oils and food by‐products, fermented preparations, polysaccharides, marine ingredients, and delivery‐enhanced products. The second maps each intervention to immune inflammation, epithelial barrier repair, pruritus and neuroimmune signaling, microbiota, metabolism, exposure, and safety. This framework distinguishes the present review from earlier catalogues and mechanism‐focused summaries in four ways. First, it explicitly connects AD phytotherapy with functional‐food and nutraceutical science, including food sources, processing parameters, bioaccessibility, and matrix effects. Second, it evaluates pediatric use and herb–drug compatibility alongside efficacy. Third, it separates association from causation in microbiome and metabolomics studies. Fourth, it grades interpretation according to evidence level and includes negative, neutral, and cautionary findings. The resulting question is not whether a plant is “natural,” but whether a chemically defined product, administered by a specified route to a defined AD population, provides clinically meaningful adjunctive benefit without weakening standard care.

## 2 AD Management: From Traditional Formulae to Target‐Resolved Bioactives

Botanical interventions for AD have developed along two linked trajectories: traditional formula‐based care and increasingly target‐resolved evaluation of extracts, dietary bioactives, and delivery systems. Recent systematic reviews and guidelines not only confirm sustained clinical interest but also show substantial heterogeneity in products, comparators, and reporting quality (Amatto et al. 2024; Limantara et al. 2024; Anheyer et al. 2025; Coyle et al. 2025; Du et al. 2025). Population studies and clinical trials indicate that patients commonly receive multicomponent formulas, topical preparations, or bathing interventions rather than isolated molecules. These data are most useful when interpreted as evidence for a defined product and use context, not as validation of all products sharing a plant name or traditional indication. Table 1 therefore separates clinical studies, preclinical extract/formula studies, and preclinical pure‐compound studies, while Table 2 appraises selected human evidence in greater detail.

**TABLE 1:** Representative clinical studies, preclinical extract/formula studies, and preclinical pure‐compound studies of botanical interventions in atopic dermatitis.

Category	Intervention/bioactive	Route/formulation	Model/population	Key finding and limitation	Refs.
Clinical studies	Chinese herbal bath therapy	External bath therapy	Pediatric AD RCTs synthesized in meta‐analysis	Pooled SCORAD/recurrence signals; regional and methodological heterogeneity	Guo et al. (2022)
Clinical studies	*Malva sylvestris* L. cream	Topical cream	Pediatric randomized double‐blind placebo‐controlled trial	Improved erythema, thickening and total SCORAD; short follow‐up	Meysami et al. (2021)
Clinical studies	Tzu‐Yun ointment	Topical ointment	Randomized open‐label comparison with topical steroid	Both groups improved; no significant between‐group superiority	Yen and Hsieh (2016)
Clinical studies	Socheongryong‐Tang	Oral multi‐herb formula	Double‐blind randomized placebo‐controlled AD trial	Trend toward reduced steroid use; broad efficacy not established	Lee, Jo, et al. (2020)
Clinical studies	Sopoongsan	Oral formula	Pilot RCT with in vitro/in vivo validation	Reduced itch NRS; small mixed AD, seborrheic population	Park et al. (2026)
Clinical studies	VGH4 standardized multi‐herb formula	Oral formula	Randomized double‐blind placebo‐controlled crossover pilot	Subjective SCORAD and quality‐of‐life signals; confirmatory trial required	Liao et al. (2026)
Clinical studies	Herbal emollient‐plus products	Topical maintenance	Adults and children with AD history; 12‐week clinical trial	Improved patient/barrier outcomes; no randomized vehicle comparator	Herrmann et al. (2025)
Clinical studies	Herbal cleanser	Topical cleanser	Randomized split‐side vehicle‐controlled pilot	TEWL improved; other biophysical outcomes inconsistent	Winayanuwattikun et al. (2023)
Clinical studies	Apple cider vinegar soaks	Topical soak	Randomized non‐blinded split‐arm pilot	No significant microbiome or *S. aureus* change	Luu et al. (2021)
Clinical studies	Natural oils/phloretin/homemade walnut cream	Topical food/cosmetic materials	Human safety reviews and reports	Barrier worsening, contact allergy or harm can occur	Karagounis et al. (2019), Gatica‐Ortega and Pastor‐Nieto (2024), Abtahi‐Naeini et al. (2023)
Preclinical studies; extracts/formulae	*Olea europaea* leaf extract + *Spirodela polyrhiza* extract	Oral combination	DNCB NC/Nga mice	Reduced dermatitis severity, IgE, histamine, Th2 signals; restored filaggrin, SIRT1, and claudin‐1	Lee et al. (2021)
Preclinical studies; extracts/formulae	Galgeunhwanggeumhwangryeon‐Tang	Topical formula	Barrier‐disruption model	Improved TEWL, pH and filaggrin; reduced KLK7, PAR2, TSLP, and IL‐4	Ahn et al. (2021)
Preclinical studies; extracts/formulae	*Schizonepeta tenuifolia*–*Saposhnikovia divaricata* decoction	Water extract	MC903‐induced AD‐like model; macrophage models; human AD tissue staining	Reduced barrier/alarmin/immune markers	Li, Liang, et al. (2024)
Preclinical studies; extracts/formulae	*Gardenia jasminoides* fruit extract	Fruit extract	Keratinocytes and 3D epidermis; MC903 mice	Restored tight‐junction factors via STAT6 regulation	Xu et al. (2026)
Preclinical studies; extracts/formulae	*Sigesbeckia pubescens* Makino extract	Oral extract	HDM NC/Nga model; stimulated skin cells	Reduced dermatitis and inflammatory markers; JAK2/STAT modulation	Song et al. (2025)
Preclinical studies; extracts/formulae	Fermented *Platycodon grandiflorum* extract	Oral fermented extract	HaCaT cells; DNCB NC/Nga mice	Altered cytokines and Th1/Th2 balance; process‐dependent candidate	Choi et al. (2021)
Preclinical studies; extracts/formulae	Fermented rice bran preparation	Oral fermented preparation	DNCB NC/Nga mice	Reduced IgE, mast cells and IL‐5/IL‐13	Saba et al. (2016)
Preclinical studies; extracts/formulae	*Sargassum serratifolium* ethanolic extract	Marine algal extract	RAW264.7, HaCaT and DNCB mice	Reduced epidermal thickness, mast cells and inflammatory signaling	Kim, Ryu, et al. (2024)
Preclinical studies; pure compounds/formulated actives	Celastrol	Isolated compound	DNFB/chronic itch models; mast cells	MRGPRX2/ORAI target‐rescue evidence; reduced scratching	Yao et al. (2023)
Preclinical studies; pure compounds/formulated actives	Glabridin liposome	Delivery‐enhanced flavonoid	Histamine‐induced model	Reduced scratching/mast‐cell activity; enhanced filaggrin	Lu, Cheng, et al. (2024)
Preclinical studies; pure compounds/formulated actives	Honokiol	Purified lignan	DNCB mice	Reduced mast cells, IgE and multiple cytokines	Lee and Im (2022)
Preclinical studies; pure compounds/formulated actives	Tea saponin	Food by‐product saponin	DNCB BALB/c model	Improved scratching, inflammation and barrier readouts	Zhang, Ma, et al. (2022)
Preclinical studies; pure compounds/formulated actives	Diphlorethohydroxycarmalol	Isolated algal phlorotannin	DNCB/HDM models and HaCaT cells	Reduced ear edema, mast cells, and cytokine/chemokine responses	Bak, Lim, et al. (2024)

**TABLE 2:** Selected human studies and translational appraisal of botanical interventions in atopic dermatitis.

Intervention	Population/design	Comparator/control	Main outcome (s)	Major limitation (s)	Refs.
Chinese herbal bath therapy	Meta‐analysis of pediatric RCTs; 8 studies and 854 children in the reported synthesis	Conventional or non‐bath controls across included trials	Improved cure rate, SCORAD, adverse reactions and recurrence in pooled analysis	Regional concentration, formula heterogeneity and trial‐quality concerns	Guo et al. (2022)
*Ficus carica* L. fruit extract cream	Randomized placebo‐controlled pediatric trial in mild‐to‐moderate AD	Vehicle placebo and 1% hydrocortisone arm	Improved SCORAD, pruritus and intensity scores over short treatment period	Small sample and short duration; botanical chemistry and long‐term safety need strengthening	Abbasi et al. (2017)
*Malva sylvestris* L. cream	Randomized double‐blind placebo‐controlled pediatric AD trial	Placebo cream	Improved thickening, erythema and total SCORAD	Short follow‐up; need vehicle characterization and relapse endpoints	Meysami et al. (2021)
Tzu‐Yun ointment	Preliminary randomized controlled open‐label trial in 33 AD patients	Topical steroid therapy	EASI and TIS decreased similarly in both arms over 8 weeks	Small, open‐label, no placebo/vehicle arm	Yen and Hsieh (2016)
Socheongryong‐Tang	Double‐blind randomized placebo‐controlled trial in AD patients with respiratory disorders	Placebo	Trend toward reduced steroid ointment dependence and acceptable short‐term safety profile	Primary signal modest; phenotype‐specific trial needs replication.	Lee, Jo, et al. (2020)
Sopoongsan	Pilot randomized placebo‐controlled trial plus translational models	Placebo	Lower itch NRS; reduced serum RANTES and IL‐4; STAT1 inhibition implicated	Only 20 participants; mixed AD/seborrheic dermatitis population	Park et al. (2026)
VGH4 standardized multi‐herb formula	Randomized double‐blind placebo‐controlled crossover pilot trial in moderate‐to‐severe AD	Placebo crossover	Subjective SCORAD and adult quality‐of‐life signals; SCORAD improvement approached significance	Pilot scale; confirmatory multicenter trial required	Liao et al. (2026)
Herbal cleanser with Acanthus, Suregada and Acacia	Double‐blind randomized split‐side vehicle‐controlled pilot in mild‐to‐moderate AD	Vehicle cleanser on contralateral side	TEWL improvement on active side	Short duration and split‐side design; other biophysical parameters less consistent	Winayanuwattikun et al. (2023)
Herbal emollient‐plus combination	12‐week clinical trial of ginger extract/cannabidiol‐containing oil‐in‐water emulsions in adults and children with AD history	No randomized vehicle comparator reported	Improved pruritus, patient‐reported outcomes, vIGA‐AD, dryness/erythema and lipid‐lamellae organization	Open/non‐comparator design limits attribution to botanical actives	Herrmann et al. (2025)
*Althaea officinalis* liposomal extract	Double‐blind randomized trial in atopic eczema	Compared side‐to‐side with steroid/placebo context in study design	Reported clinical improvement without obvious side effects in short‐term use	Small trial; delivery system and comparator details need careful reporting	Khalighi et al. (2021)
Apple cider vinegar soaks	Randomized non‐blinded split‐arm pilot microbiome study in AD and healthy controls	Tap‐water control arm	No significant alteration of skin bacterial microbiome or *S. aureus* abundance after 14 days	Small, non‐blinded, concentration‐specific study	Luu et al. (2021)

**FIGURE 1:**
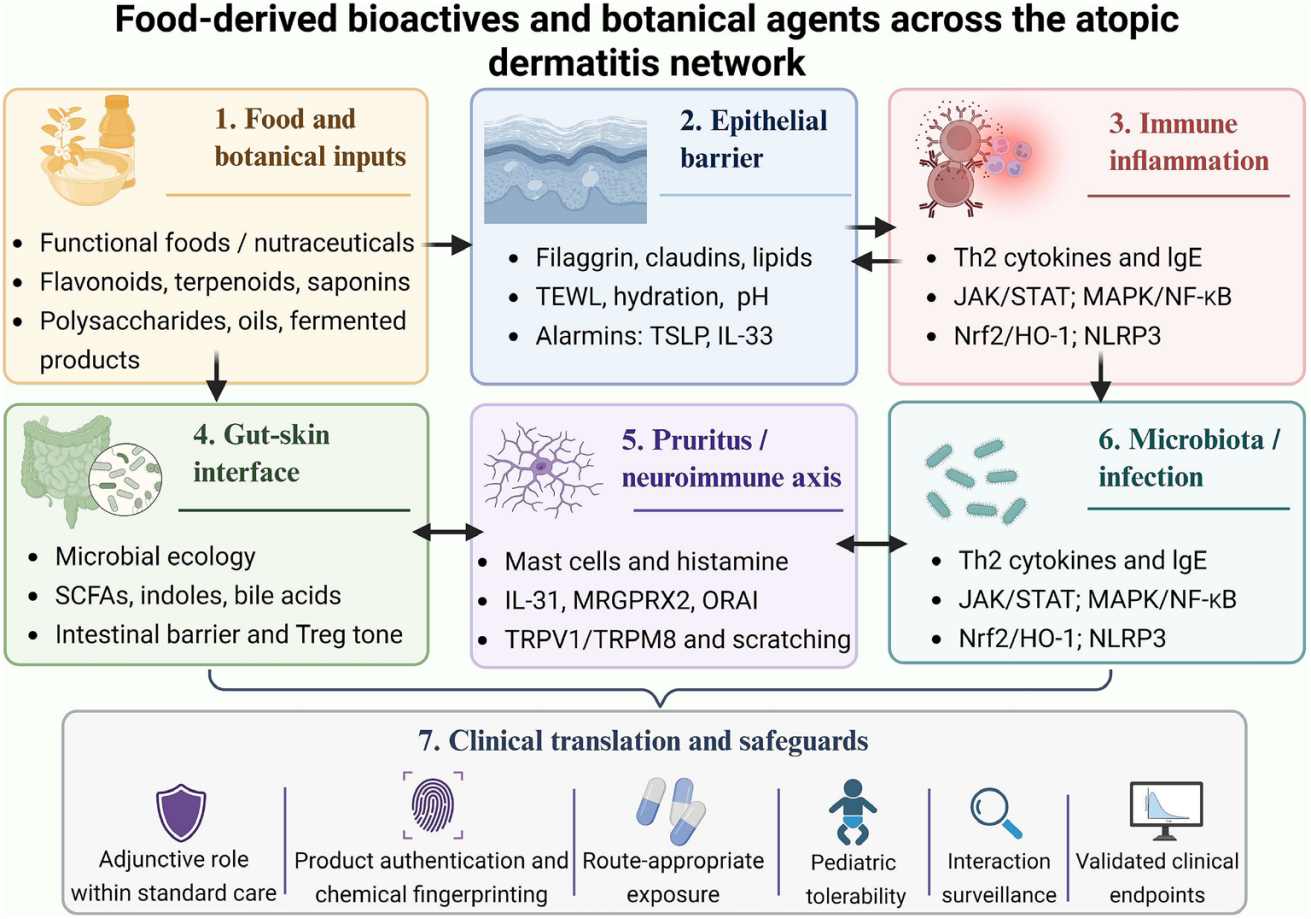
Conceptual model linking food‐derived bioactives and botanical agents to major atopic dermatitis axes. Candidate interventions may influence epithelial barrier integrity, immune inflammation, pruritus and neuroimmune signaling, the gut‐skin interface, and skin microbiota or infection‐related pathways. Route‐appropriate exposure encompasses oral bioavailability and topical delivery systems. Clinical translation requires adjunctive use, product standardization, pediatric tolerability, interaction surveillance, and validated clinical endpoints.

Experimental studies provide mechanistic depth but are dominated by DNCB/DNFB, oxazolone, MC903, and house‐dust‐mite models supported by keratinocyte, mast‐cell, and macrophage systems. Many preparations reduce erythema, ear thickness, IgE, mast‐cell infiltration, and Th2 cytokines; fewer establish exposure, target engagement, pathway necessity, or durable relapse prevention. Accordingly, repeated improvement in an acute sensitization model is treated here as preclinical support, not clinical efficacy. Greater weight is assigned to studies that define the extract chemically, use dose–response designs, include rescue or perturbation experiments, reproduce effects across models, or validate findings in human‐relevant skin systems (Yao et al. 2023; Li, Liang, et al. 2024; Lu, Deng, et al. 2024; Yang, Jeong, et al. 2025; Yang, Zhou, et al. 2025). A rigorous synthesis must consider product identity, route, comparator quality, age, disease severity, background therapy, follow‐up, rescue medication, and adverse‐event reporting. It must also preserve negative evidence. Apple cider vinegar soaks did not significantly modify the skin microbiome in a small split‐arm study; Tzu‐Yun ointment improved scores without outperforming topical steroid therapy; an herbal emollient‐plus study lacked a randomized vehicle comparator; olive oil may aggravate barrier dysfunction in some settings; and phloretin or homemade food‐based preparations can cause contact allergy or clinical deterioration (Yen and Hsieh 2016; Luu et al. 2021; Herrmann et al. 2025; Karagounis et al. 2019; Gatica‐Ortega and Pastor‐Nieto 2024; Abtahi‐Naeini et al. 2023). These findings counter efficacy‐centered publication bias and define where product development should stop, be reformulated, or proceed only under controlled conditions. The major mechanism‐level modules linking botanical interventions to AD pathobiology, including immune inflammation, epithelial alarmins, barrier restoration, pruritus, oxidative stress, inflammasome activation, microbiota, gut‐skin signaling, and delivery science, are summarized in Figure 1.

## 3 Oral, Topical, and Bathing Formulae: Clinical Signals and Unresolved Trial‐Design Problems

Chinese herbal medicine and related East Asian formulae dominate the clinical evidence. Their use is frequently justified by chronicity, steroid concerns, and the need for relapse prevention, but the evidence is not uniform. A database study showed that pediatric TCM users often had comorbid allergic rhinitis and that Chinese herbal medicines were prescribed in multicomponent combinations, with Xiao‐Feng‐San functioning as a core network node (Chen et al. 2016). These studies are not efficacy trials, but they are important because they identify what actually enters clinical practice: not isolated molecules, but repeatable formula clusters shaped by syndrome patterns, comorbidity, and prior corticosteroid exposure. Randomized or controlled clinical studies provide more direct but still limited evidence. Tzu‐Yun ointment was compared with topical steroid therapy in a preliminary randomized open‐label study and produced reductions in EASI and TIS without significant between‐group differences over 8 weeks (Yen and Hsieh 2016). Sopoongsan was evaluated in a randomized, double‐blind, placebo‐controlled pilot trial for chronic upper‐body pruritus diagnosed as atopic or seborrheic dermatitis, with reductions in itch intensity and serum RANTES/IL‐4 trends supported by translational STAT1 work (Park et al. 2026). Socheongryong‐Tang showed a tendency toward reduced steroid ointment dependence and no marked safety abnormalities in AD patients with respiratory disorders (Lee, Jo, et al. 2020). These findings are clinically interesting because they target not only eczema severity, but also itch, respiratory comorbidity and steroid‐sparing behavior.

Topical and bathing interventions are particularly relevant for children because they can theoretically deliver local benefit with lower systemic exposure. The pediatric herbal bath meta‐analysis reported superiority over controls in cure rate, SCORAD, adverse reactions and recurrence, although the strength of inference is limited by trial quality and regional concentration (Guo et al. 2022). *Malva sylvestris* cream, Jaungo, an herbal cleanser, herbal emollient‐plus products, and the BSZY cream protocol illustrate the shift from traditional topical use toward products that can be evaluated against vehicle controls or standard topical agents (Herrmann et al. 2025; Meysami et al. 2021; Winayanuwattikun et al. 2023; Ahn et al. 2018). The BSZY trial protocol is especially notable because it specifies a randomized double‐blind controlled design, facial AD target population, SCORAD primary endpoint, and safety monitoring (Wang et al. 2025). The clinical literature also includes integrative and combination strategies. Herbal medicines combined with Western medicines, Jianpi formulas, Longmu Tang granules, Huoxiang Zhengqi/Gwakhyangjeonggi‐san, and Qin‐Zhu‐Liang‐Xue decoction reflect a pragmatic clinical reality: Botanicals are frequently added to, rather than substituted for, standard care (Kim and Kim 2016; Huang, Liu, et al. 2025; Li, Shen, et al. 2022; Li, Sun, et al. 2022; Xuan et al. 2021; Son et al. 2020). This is a strength if it reduces disease burden, improves adherence, or decreases rescue medication; it is a weakness if trials cannot separate botanical efficacy from background treatment, natural disease fluctuation, or placebo effects. Future trials should prespecify whether the botanical intervention is intended as monotherapy, steroid‐sparing adjunct, relapse‐prevention maintenance, itch‐directed rescue, or barrier‐support co‐treatment.

Several apparently promising interventions also illustrate why biological activity or within‐group improvement may not translate into persuasive clinical benefit. Tzu‐Yun ointment reduced EASI and TIS but did not outperform topical corticosteroid therapy; apple cider vinegar soaks did not significantly alter the skin microbiome or *S. aureus* abundance; and the herbal emollient‐plus study lacked a randomized vehicle comparator, preventing attribution of benefit to the botanical ingredients (Yen and Hsieh 2016; Luu et al. 2021; Herrmann et al. 2025). Neutral or equivocal outcomes may reflect insufficient target‐site exposure, unstable or poorly standardized composition, nonspecific vehicle effects, heterogeneous AD endotypes, insensitive endpoints, short follow‐up, or strong background‐treatment effects. Such findings should be used to refine candidate selection, dose, formulation, and trial design rather than being excluded from efficacy‐centered summaries.

The main trial‐design problems are recurrent. Placebo construction is difficult because decoctions, ointments, and baths have odor, color, texture, and sensory effects. Blinding is often incomplete. Formula composition may vary by practitioner, batch, supplier, or syndrome differentiation. Outcomes range from SCORAD and EASI to local symptom scores, pruritus scales, topical steroid use, and vague global improvement, making meta‐analysis unstable. Follow‐up is often shorter than the relapse cycle that matters to patients. Reporting of botanical dermatology interventions has historically been incomplete, including insufficient intervention and safety details (Lev‐Tov et al. 2016). Pediatric trials should additionally prespecify age‐stratified dosing, growth, and developmental safety capture, caregiver‐reported sleep endpoints, and standardized adverse‐event coding. These gaps are solvable, but only if botanical AD trials adopt the same methodological expectations now applied to biologics, JAK inhibitors, and nonsteroidal topical agents. Formula standardization is particularly difficult because traditional prescribing often allows individualized modifications. A strict pharmaceutical trial prefers one fixed formula, one dose, and one schedule. A traditional clinical logic may prefer syndrome‐adapted formulae, seasonal adjustments, or symptom‐guided modification. Both approaches can be studied, but they answer different questions. Fixed‐formula trials test a product; individualized trials test a treatment strategy. A mature evidence base should include both, but the protocol must state which is being evaluated and should report the actual formula exposure received by each participant. Another unresolved issue is steroid‐sparing interpretation. Reduced topical steroid use may reflect true disease improvement, caregiver preference, fear of steroids, or inadequate rescue treatment. Therefore, steroid‐sparing should never be interpreted in isolation. It should be paired with validated severity scores, flare counts, itch and sleep outcomes, photographs assessed by blinded dermatologists, and predefined rescue criteria.

## 4 Active Constituents and Extract Classes: From Ethnobotanical Complexity to Pharmacological Modules

### 4.1 Food Sources and Nutritional Context

Many candidate AD bioactives originate from foods, beverages, or edible processing streams, but the relevant source and exposure differ substantially. Puerarin is concentrated in kudzu (Pueraria) root, which is also processed into starch‐containing foods; luteolin occurs in celery, parsley, peppers, and several Brassica vegetables. Tea saponins can be recovered from *Camellia oleifera* seed pomace; blackcurrant supplies complex polysaccharides; and rice bran, soybean, sea‐buckthorn oil, and edible algae provide food‐derived fractions relevant to immune or barrier biology. Source attribution matters because a purified compound, a concentrated extract, and a whole food do not deliver equivalent doses or co‐constituents. Accordingly, “food‐derived” should be interpreted as a provenance descriptor rather than as proof of oral efficacy or safety. Flavonoids and related polyphenols include puerarin, luteolin derivatives, glabridin, diosmetin, naringenin, protocatechuic aldehyde, mangiferin, aspalathin, oroxylin A, and rosmarinic acid‐like compounds. Terpenoids include honokiol, bisabolol, celastrol, sarsasapogenin‐like molecules, and essential‐oil components. Saponins and glycosides include tea saponin, tomato saponins, lycoperoside H, platycodin‐related compounds, and mogroside‐containing extracts. Polysaccharides, fatty acids, and marine lipids form additional classes. This chemical organization is not merely taxonomic: Different classes tend to cluster around different AD axes, such as JAK/STAT signaling for selected flavonoids, mast‐cell degranulation for terpenoids, and barrier lipid/TEWL recovery for oils and marine lipids (Wu et al. 2021). Flavonoid‐rich interventions are among the most mechanistically coherent. Puerarin attenuated DNCB‐induced AD‐like lesions and inhibited inflammatory cytokines and chemokines in HaCaT cells (Lee et al. 2018). Luteolin 7‐O‐glucoside from *Stellera chamaejasme* reduced serum IgE and IL‐4, improved transepidermal water loss, and increased hydration (Jo et al. 2019). Luteolin 7‐methyl ether from *Wikstroemia ganpi* inhibited TNF‐α/IFN‐gamma‐induced inflammatory signaling in keratinocytes (Jegal et al. 2021). Protocatechuic aldehyde targeted STAT3‐JAK1 interaction in epidermal inflammation (Yang, Zhou, et al. 2025), and aspalathin restored STAT6‐mediated immune dysregulation (Yang, Jeong, et al. 2025). These results suggest a recurring flavonoid logic: suppression of cytokine‐driven keratinocyte activation, inhibition of STAT‐dependent Th2 amplification, and partial restoration of barrier‐associated proteins.

### 4.2 Processing, Fermentation, and Stability

Industrial and traditional processing can enhance, diminish, or redirect biological activity. Cell disruption, milling, and controlled heating may release matrix‐bound phenolics or inactivate oxidative enzymes, while excessive temperature, oxygen, or storage can degrade flavonoids, oxidize lipids, and alter sensory or irritancy profiles. In apple matrices, thermal and mechanical processing reduced total polyphenols but increased their digestive bioaccessibility, illustrating why concentration and accessible dose can move in opposite directions (Alongi et al. 2025). Microwave processing of *Hydrangea macrophylla* increased hydrangenol generation and anti‐AD activity, whereas aging of Camellia oil changed chemical and biological properties (Kim, Ju, et al. 2022; Ouyang et al. 2024). These examples support direct measurement rather than an assumption that “processed” means weaker or that “fermented” means stronger.

Fermentation efficacy is parameter‐dependent. Microbial strain controls the enzyme repertoire available for deglycosylation, decarboxylation, hydrolysis, and *de novo* metabolite production; time determines whether desirable conversion reaches a plateau or proceeds to degradation; temperature governs growth, enzyme activity, and oxidation; and pH changes substrate solubility, microbial succession, and chemical stability. Substrate‐to‐water ratio, oxygen transfer, inoculum size, and downstream pasteurization or drying further influence the final composition. Studies of fermented rice bran and *Platycodon grandiflorum* support bioconversion as a plausible source of altered activity, but they do not establish a universal fermentation effect (Saba et al. 2016; Choi et al. 2021). Future work should report the starter strain at strain level, inoculum, fermentation time course, temperature, pH trajectory, oxygen conditions, yield, pre/post‐fermentation fingerprints, and stability after drying or storage.

### 4.3 Oral Bioavailability and Food‐Matrix Effects

For orally administered bioactives, nominal content is only the first step in exposure. Bioaccessibility requires release from the food or extract matrix during digestion; absorption then depends on solubility, micellar incorporation, intestinal transport, and first‐pass metabolism. Polyphenols bound to fiber or cell wall structures may be less accessible, whereas processing or co‐ingested lipids can increase release or micellarization. Protein, pectin, and other polysaccharides can bind phenolics and alter both upper gastrointestinal absorption and the fraction delivered to the colon (Tarko and Duda‐Chodak 2020; García‐Pérez et al. 2024; Tomas et al. 2024). Thus, the same purified compound, capsule, beverage, and whole‐food matrix may produce different parent‐compound and microbial‐metabolite exposure. This distinction is important for AD because oral efficacy may be mediated by absorbed parent compounds, conjugated metabolites, microbial products, or nonspecific nutritional changes. Claims for an oral functional food or nutraceutical should therefore include standardized dose, in vitro digestion or bioaccessibility data, plasma or urinary exposure when feasible, and separation of matrix effects from the active ingredient. Conversely, topical products avoid gastrointestinal losses but face stratum‐corneum penetration, chemical stability, and local tolerability constraints.

## 5 Immune Inflammation: Th2 Dominance, Epithelial Alarmins, and Intracellular Signaling

Botanical and plant‐derived interventions in AD most consistently converge on attenuation of type 2 inflammation, although the underlying immunological mechanisms vary across extracts, formulae, and experimental systems. In DNCB/DNFB‐, oxazolone‐, MC903‐, and house‐dust‐mite–based models, many preparations reduce IL‐4, IL‐5, IL‐13, serum IgE, eosinophil infiltration, CD4‐positive T‐cell activation, IgE‐producing B‐cell markers, mast‐cell accumulation, and pruritus‐associated inflammatory mediators (Cho et al. 2017; Moon and Kim 2020; Yao et al. 2023; Bak, Park, et al. 2024; Jo et al. 2019). *Olea europaea* leaf plus *Spirodela polyrhiza* reduced serum IgE, histamine, and Th2 cytokines while increasing IFN‐gamma in splenocytes, suggesting partial correction of Th2 bias (Lee et al. 2021). *Paeoniae radix* alba root extract reversed Th2 skewing as shown by IgE, IgG1/IgG2a ratio, and IL‐4/IFN‐gamma balance (Jo et al. 2018). *Aristotelia chilensis* water extract increased IFN‐gamma and decreased IL‐4 in spleen cells (Moon and Kim 2020). These studies support the claim that certain plant extracts can modulate Th1/Th2 balance, but they do not establish a uniform immunological mechanism. A more refined pathophysiological framework is provided by epithelial alarmins. In human AD, keratinocyte‐derived TSLP, IL‐33, and IL‐25 promote type 2 immunity, itch, and dendritic‐cell activation. Several botanical studies measure TSLP and IL‐33, connecting barrier injury to immune amplification. Galgeunhwanggeumhwangryeon‐Tang reduced KLK7, PAR2, TSLP, and IL‐4 while increasing filaggrin in a barrier‐disruption model (Ahn et al. 2021). *Gardenia jasminoides* fruit extract reduced IL‐4/IL‐13‐driven tight‐junction disruption through STAT6 regulation (Xu et al. 2026). *Lobelia chinensis* and diosmetin reinforced barrier function through SPINK5/LEKTI‐related effects and attenuated alarmin‐linked inflammation (Park et al. 2022). *Dictamnus dasycarpus* root bark improved barrier function and symptoms while influencing TSLP and inflammatory pathways (Park et al. 2024).

Intracellular signaling studies further place botanical AD research within the therapeutic logic of contemporary dermatology. JAK/STAT signaling is one of the clearest points of convergence between botanical research and current AD therapeutics. Modern small‐molecule JAK inhibitors have established that cytokine signaling through JAK1, JAK2, STAT3, STAT6, and related nodes is clinically actionable. Plant‐derived interventions are not JAK inhibitors in the pharmaceutical sense, but several studies report modulation of STAT1, STAT3, or STAT6 pathways. Sopoongsan linked antipruritic effects to STAT1 inhibition (Park et al. 2026). *Sigesbeckia pubescens* suppressed JAK2/STAT signaling in stimulated skin cells and restored barrier function in house dust mite‐treated NC/Nga mice (Song et al. 2025). Lagerstroemia macrocarpa inhibited Th2‐mediated STAT6 signaling pathway in human keratinocytes (Seo et al. 2024). Protocatechuic aldehyde disrupted STAT3‐JAK1 interaction (Yang, Zhou, et al. 2025), and aspalathin restored STAT6‐mediated immune dysregulation (Yang, Jeong, et al. 2025).

MAPK/NF‐κB signaling is even more common. Chijabyukpi‐Tang, bisdemethoxycurcumin, albizia extract, bisabolol, *Moringa concanensis*, *Polygonum perfoliatum* , *Viola yedoensis*, Derma‐Hc, black soybean extract, and mangiferin all report inhibition of inflammatory signaling linked to MAPK, NF‐κB, JNK, p38, or AP‐1 (Lee, Lim, et al. 2020; Wang et al. 2023; Kim, Jin, et al. 2025; Kim, Kim, et al. 2022; Li, Wu, et al. 2022; Fan et al. 2024, 2021; Nam et al. 2021; Dorjsembe et al. 2022; Lu, Deng, et al. 2024). This recurrence is plausible because many polyphenols and terpenoids can influence oxidative stress and inflammatory kinase cascades. The limitation is specificity: Inhibition of NF‐κB in a stimulated cell line is a general anti‐inflammatory signal, not an AD‐specific mechanism. To elevate such findings, future studies should connect pathway modulation to disease‐relevant upstream triggers, such as IL‐4/IL‐13, TSLP, *S. aureus* products, barrier lipids, or itch‐associated neurons. Nrf2/HO‐1 and NLRP3 inflammasome pathways add two additional immune‐metabolic axes. Chijabyukpi‐Tang increased Nrf2 nuclear translocation and HO‐1 expression while improving DNCB‐induced lesions (Lee, Lim, et al. 2020). *Eclipta prostrata* attenuated HDM‐induced AD‐like inflammation by restoring barrier function and suppressing ERK/STAT1 and NF‐κB‐associated signaling (Kang et al. 2022). *Moringa concanensis* attenuated NLRP3 inflammasome‐mediated IL‐1β activation, with improvement in dermatitis score, ear thickness, and transepidermal water loss (Kim, Kim, et al. 2022). Advanced nanocarrier and carbon‐nanodot studies broaden delivery options, but they do not by themselves establish inflammasome‐specific mechanisms (Vyas et al. 2026; Jiang et al. 2026). Together, these pathways broaden botanical AD research beyond a purely Th2‐centered model and suggest that oxidative stress, innate immune activation, and intracellular signal integration may sustain chronic disease activity.

This immune literature nevertheless requires careful interpretation. Many studies quantify broad cytokine panels, but fewer distinguish primary target engagement from secondary changes that follow reduced scratching, improved barrier integrity, or lower dermatitis severity. A fall in IgE, IL‐4, IL‐13, or IL‐31 after treatment is informative, but it does not prove that the measured cytokine is the direct pharmacological target. Mechanistic confidence increases when studies include pathway rescue, overexpression, pharmacological inhibition, target‐engagement assays, time‐course sampling, or validation in human tissue. In this regard, celastrol reversal by MRGPRX2 overexpression, macrophage TRPV1 work in JF decoction, STAT‐focused studies, and target‐specific flavonoid experiments represent stronger designs (Yao et al. 2023; Li, Liang, et al. 2024; Yang, Zhou, et al. 2025; Lu, Deng, et al. 2024; Yang, Jeong, et al. 2025). The alarmin‐barrier loop is especially important for botanicals because many candidate products affect both sides of the loop. Barrier damage promotes TSLP and IL‐33 release; these alarmins intensify type 2 immunity; type 2 cytokines suppress epidermal differentiation; barrier damage worsens further. A product that slightly improves filaggrin while slightly reducing STAT6 signaling could, in theory, produce a larger clinical effect than either action alone. This is the most plausible biological rationale for multicomponent botanical products, but it must be tested with time‐course experiments that distinguish early target engagement from late disease improvement. Endotype specificity remains underexplored. Pediatric extrinsic AD with high IgE, adult chronic lichenified AD, Asian AD with stronger Th17/Th22 signals, facial dermatitis with cosmetic exposure, and infection‐prone AD may not respond to the same botanical intervention. Most preclinical studies use one model and one mouse strain. Future work should match candidate products to endotypes: IL‐31/TRP‐directed products for itch‐dominant disease, lipid‐rich products for xerotic barrier failure, antimicrobial or microbiome‐modulating products for *S. aureus* ‐dominant flares, and STAT6‐directed flavonoids for type 2‐high inflammation.

## 6 Barrier Restoration: Filaggrin, Tight Junctions, Lipids, and Stratum‐Corneum Physiology

Epidermal barrier dysfunction is a central pathogenic component of AD rather than a passive consequence of inflammation. Defective barrier integrity promotes allergen penetration, microbial colonization, epithelial alarmin release, itch amplification, and recurrent inflammation; therefore, botanical interventions that restore barrier structure may provide broader clinical benefit than agents that only suppress inflammatory mediators. Across experimental AD models, several plant‐derived preparations have been associated with increased filaggrin, claudin‐1, SIRT1, involucrin‐related differentiation markers, skin hydration, and reduced transepidermal water loss (TEWL). The *Olea europaea* /Spirodela combination restored filaggrin, SIRT1, and claudin‐1 (Lee et al. 2021). Galgeunhwanggeumhwangryeon‐Tang recovered skin‐lipid barrier function and increased filaggrin while reducing TSLP/IL‐4 signaling (Ahn et al. 2021). *Siraitia grosvenorii* residual extract increased claudin‐1, SIRT1, and filaggrin in lesional skin (Sung et al. 2020). *Gardenia jasminoides* extract and fruit preparations repeatedly connect barrier recovery with STAT6 and MAPK‐related pathways (Xu et al. 2026). However, filaggrin restoration alone is not sufficient to define barrier repair. AD barrier failure also involves corneodesmosome proteases, lipid lamellae, ceramide composition, tight‐junction integrity, antimicrobial peptides, skin pH, and mechanical damage caused by scratching. In this context, herbal emollient‐plus studies that include instrumental evaluation of intercellular lipid content and lipid lamellae organization represent an important methodological advance (Herrmann et al. 2025). Lipid‐rich natural products, including *Macrocystis pyrifera* lipids and sea buckthorn oil, further suggest that selected botanical or marine‐derived lipid fractions may directly influence cytokine‐induced barrier dysfunction and stratum‐corneum repair (Kok et al. 2023; Wang et al. 2020).

Tight‐junction regulation provides a particularly important mechanistic bridge between immune inflammation and epithelial repair, especially in facial and pediatric AD, where thin skin, frequent irritant exposure, and cosmetic or topical‐product use complicate management. *Gardenia jasminoides* fruit extract reduced IL‐4/IL‐13‐induced tight‐junction disruption by regulating JAK‐STAT6 signaling pathway (Xu et al. 2026). *Pulsatilla koreana*, *Glycine soja*, and related flavonoid‐rich extracts also report barrier‐factor regulation through JAK/STAT and inflammatory pathways (Kim, Park, et al. 2025; Sung et al. 2025). Agerarin promoted filaggrin recovery through JAK/STAT‐linked mechanisms (Ahn et al. 2020). These findings support a dual model of botanical barrier repair: Some plant‐derived molecules may restore epidermal proteins by preventing Th2 cytokine‐mediated suppression of keratinocyte differentiation, whereas others may act more directly on keratinocyte maturation, lipid metabolism or structural barrier assembly. This distinction is clinically important because a barrier‐directed botanical product may serve different roles depending on its dominant activity: rapid symptom relief during flares, adjunctive anti‐inflammatory support, or long‐term maintenance of stratum‐corneum function. Barrier repair also has a temporal dimension. Early reductions in TEWL may reflect transient occlusion or humectancy rather than durable structural remodeling, whereas longer application periods may be required to normalize lipid lamellae, differentiation markers, and relapse susceptibility. A 4‐week trial may detect itch or erythema improvement but miss flare‐prevention effects; conversely, a 12‐week emollient‐plus study may reveal progressive lipid lamella restoration that would not be captured in shorter designs (Herrmann et al. 2025). Future studies should therefore distinguish immediate moisturizing effects from sustained barrier remodeling by measuring endpoints before application, after washout and during follow‐up.

Topical oils, emollients, and food‐derived preparations require particularly careful interpretation because “natural” does not necessarily mean barrier‐restorative or non‐irritating. Olive oil, coconut oil, and sunflower seed oil differ in oleic/linoleic acid content, irritancy, and effects on TEWL, and olive oil may worsen xerosis in some contexts (Karagounis et al. 2019). Home preparations can also cause complications, as illustrated by infant AD complicated by homemade walnut cream (Abtahi‐Naeini et al. 2023). Product design must therefore distinguish between occlusion, humectancy, lipid replacement, anti‐inflammatory phytochemicals, and irritant or allergenic residues. This distinction is particularly important in pediatric AD, where topical exposure occurs on a compromised barrier and where food‐derived allergens, fragrance constituents, or poorly defined plant residues may increase sensitization risk. Barrier‐centered botanical interventions are attractive because they can align with first‐line AD care rather than compete with it. Moisturizers are recommended across AD severities, and a standardized botanical emollient‐plus product could be evaluated as an enhanced moisturizer, steroid‐sparing maintenance agent, or flare‐prevention adjunct (Sidbury et al. 2023). Such claims, however, should be anchored to measurable barrier endpoints, including TEWL, hydration, pH, lipidomics, Raman or tape‐stripping biomarkers, filaggrin degradation products, microbial colonization, and patient‐reported dryness. Anatomical site should also be considered: Facial AD, hand AD, flexural AD, and generalized xerosis differ in barrier thickness, irritant exposure, cosmetic use, social burden, and tolerability requirements. A botanical cream tolerated on the trunk may sting on the face, whereas bath therapy may be useful for widespread pediatric eczema but impractical for localized eyelid dermatitis. Site‐specific efficacy and tolerability should therefore be reported rather than assuming that a whole‐body dermatitis score captures all clinically meaningful barrier outcomes.

## 7 Pruritus, Mast Cells, and Neuroimmune Signaling

Pruritus is one of the most clinically consequential manifestations of AD because it drives sleep disruption, excoriation, secondary infection risk, and sustained impairment in quality of life. Botanical and plant‐derived interventions may be particularly relevant to this dimension of disease when they modulate mast‐cell activation, histamine release, IL‐31 signaling, sensory‐neuron excitability, or transient receptor potential channels. *Diospyros lotus* leaf and grapefruit stem extract reduced mast‐cell infiltration, serum IgE, IL‐4, and scratching behavior in AD‐like mice (Cho et al. 2017). Celastrol suppressed MRGPRX2/ORAI‐axis activation, histamine release, and scratching behavior, with mechanistic reversal after MRGPRX2 overexpression (Yao et al. 2023). Glabridin liposomes reduced histamine‐induced scratching, mast‐cell infiltration/degranulation, and nerve growth factor while increasing filaggrin (Lu, Cheng, et al. 2024). Mast‐cell degranulation is a recurring target across extracts and compounds. *Pterocarpus santalinus* inhibited β‐hexosaminidase and histamine release in IgE‐sensitized mast cells and improved DNCB‐induced lesions (Ham et al. 2019). (‐)‐α‐Bisabolol inhibited JNK and NF‐κB in mast cells (Li, Wu, et al. 2022). Honokiol, *P. radix* alba, fermented rice bran, date‐plum leave extracts, *Albizia julibrissin* , vasicine, *Callerya atropurpurea*, and *Callicarpa dichotoma* all report reductions in mast‐cell infiltration, degranulation, or mast‐cell‐linked cytokines (Jo et al. 2018; Saba et al. 2016; Lee and Im 2022; Cho et al. 2020; Kim, Jin, et al. 2025; Zhang, Du, et al. 2022; Choi et al. 2023; Kim, Lee, et al. 2022). These findings provide a plausible basis for botanical antipruritic activity, although reduced mast‐cell infiltration in murine dermatitis models may sometimes reflect overall attenuation of inflammation rather than direct mast‐cell targeting.

More disease‐specific insight emerges from studies addressing IL‐31 and broader neuroimmune itch circuits. Modern AD itch is increasingly understood as a dynamic interaction among keratinocytes, immune cells, mast cells, macrophages, sensory neurons, and scratching‐induced barrier trauma, rather than as a simple consequence of histamine release. Sopoongsan, Herba Siegesbeckiae, *Ampelopsis brevipedunculata* , fraxinellone, *Viola yedoensis*, hydrolyzed celery extract, and several formulae explicitly measure IL‐31 or itch‐related endpoints, placing botanical interventions within this more contemporary neuroimmune framework (Park et al. 2026; Bak, Park, et al. 2024; Wang et al. 2026; Yang et al. 2024; Che et al. 2020; Fan et al. 2021). Mechanistic studies targeting sensory pathways further extend this concept. JF decoction targeting macrophage TRPV1 and *Agrimonia coreana* extract inhibiting CRAC/ORAI‐mediated calcium signaling extend the itch framework beyond conventional histamine pathways (Li, Liang, et al. 2024; Kim, Lee, et al. 2024). This neuroimmune perspective has important implications for clinical interpretation. A botanical product may produce only modest improvement in EASI or erythema while still delivering meaningful benefit if it reduces nocturnal itch, scratching frequency, and sleep fragmentation. Conversely, a compound that lowers IL‐4 or IgE in experimental models but fails to improve human itch or sleep may have limited practical value as an antipruritic intervention.

Clinical development of itch‐directed botanical interventions should therefore prioritize endpoint rigor, tolerability, and formulation safety. Scratching counts in mice are useful for mechanistic screening, but they are not equivalent to human itch intensity, sleep impairment, caregiver‐observed nocturnal scratching, or itch‐related quality of life. Human studies should include peak pruritus numerical rating scale, sleep disturbance, patient‐oriented eczema measures, actigraphy when feasible, rescue medication use, and adverse sensory symptoms. Biomarkers such as serum IL‐31, lesional nerve growth factor, or mast‐cell mediators should be paired with clinical itch outcomes rather than used as substitutes for them. This is particularly important in children, where caregiver‐reported sleep disruption, nocturnal awakenings, excoriation burden, and scratching behavior may be more informative than generic dermatology quality‐of‐life scales alone. Safety assessment is equally important because agents that activate cold receptors, modulate TRP channels, or contain essential‐oil terpenoids may produce burning, stinging, paradoxical irritation, or irritant dermatitis, especially on compromised AD skin. Products designed for itch relief should not aggravate barrier injury. The most defensible translational design would be a short‐term add‐on trial in stable mild‐to‐moderate AD, using standardized moisturizer background therapy, defined rescue topical anti‐inflammatory rules, blinded vehicle control, and a primary itch endpoint supported by TEWL, sleep, and adverse‐sensation monitoring. An ideal antipruritic botanical formulation should be chemically defined, fragrance‐minimized, non‐sensitizing, and tested on impaired skin rather than only on intact volunteer skin.

## 8 Microbiota, Metabolism, and the Host–Environment Interface

The gut‐skin axis is a plausible route through which dietary bioactives may influence AD, but evidence must be separated by causal strength. Cross‐sectional dysbiosis can be a consequence of diet, antibiotics, age, disease severity, or treatment. More informative longitudinal data show that selected early‐life gut microbial differences can precede physician‐diagnosed eczema, although temporal precedence does not by itself prove causation (Cheung et al. 2023). Experimental transfer studies provide a stronger perturbational step: Fecal microbiota transplantation (FMT) from healthy donors improved AD‐like phenotypes in OVA‐ or allergen‐based juvenile mouse models, supporting microbiota‐dependent modulation while remaining limited by model and donor effects (Kim et al. 2021; Wang et al. 2024). Accordingly, the gut microbiome is presented as a mediator or effect modifier, not a universally established primary cause of AD.

Food‐derived polysaccharides, fermentable fibers, and phytochemicals may alter substrate availability, microbial community structure, and production of short‐chain fatty acids, indoles, phenolic metabolites, and secondary bile acids. These metabolites can influence intestinal epithelial integrity, dendritic‐cell and regulatory‐T‐cell tone, systemic type 2 inflammation, and potentially epidermal lipid or barrier responses. Fermented rice bran, fermented Platycodon, blackcurrant polysaccharide, *Paeonia lactiflora*, and Houttuynia polysaccharides provide candidate links between oral exposure, microbial or metabolic change, and AD‐like outcomes (Saba et al. 2016; Choi et al. 2021; Ashigai et al. 2018; Lee et al. 2022; Huang, Zhang, et al. 2025). However, most studies do not show that the microbiota change is necessary for efficacy. Antibiotic depletion, gnotobiotic transfer, metabolite supplementation, or receptor blockade is needed to move from correlation to mechanism. Causal direction is likely bidirectional. AD can alter sleep, diet, stress, antimicrobial exposure, and systemic inflammation, all of which can modify the gut microbiome; microbial metabolites may in turn alter immune maturation and barrier function. Future clinical studies should therefore use repeated pre‐treatment sampling, record diet and antibiotics, distinguish incident from established AD, and integrate stool metagenomics with measured metabolites and skin endpoints. A convincing dietary‐bioactive mechanism would require a defined product, a reproducible microbial or metabolite shift, temporal mediation of an AD endpoint and, ideally, perturbation or rescue evidence.

Metabolic regulation provides a second host interface mechanism through which botanical products may influence AD. The epidermis is an active lipid and amino‐acid metabolic tissue, and AD severity is shaped by ceramide composition, free fatty acids, cholesterol balance, filaggrin‐derived natural moisturizing factors, oxidative stress, and inflammatory lipid mediators. Herba Siegesbeckiae metabolomics identified differential metabolites, including amino acid, glycerophospholipid, histidine, and prostaglandin‐related pathways, as putative mediators of anti‐AD activity (Wang et al. 2026). A topical mixture of *Asarum sieboldii*, *P. grandiflorum* , and *Cinnamomum cassia* extracts shifted serum and lesional skin metabolites related to fatty acid metabolism, oxidative stress, and immune regulation (Lee, Jung, et al. 2024). Additional lipid‐ or redox‐oriented interventions, including aged *C. oleifera* oil, *Macrocystis* lipids, *Schisandra chinensis*, sea buckthorn oil, and Nrf2/HO‐1‐linked products, further support the idea that botanical preparations may be particularly relevant at the intersection of inflammation, oxidative stress, and barrier metabolism (Ouyang et al. 2024; Kok et al. 2023; Son et al. 2023; Wang et al. 2020; Kang et al. 2022). However, metabolomic data require cautious interpretation. Metabolomics can reveal exposure‐responsive pathways but cannot establish function on its own. A list of altered metabolites does not establish mechanism unless the changes are linked to dose, timing, tissue localization, functional rescue, or patient‐relevant barrier and inflammatory outcomes.

The host–environment interface further extends beyond microbiota and metabolism to include pollutants, detergents, bathing practices, preservatives, cosmetics, and repeated topical exposure. Plant‐derived products may be protective, neutral, or harmful depending on formulation, concentration, vehicle, anatomical site, and barrier status. Antimicrobial phytochemicals that inhibit *S. aureus* growth, biofilm formation, or quorum sensing are conceptually relevant, but direct clinical evidence in AD remains limited. Conversely, phloretin contact dermatitis in a patient with AD demonstrates that cosmetic‐grade natural compounds can cause allergic contact dermatitis (Gatica‐Ortega and Pastor‐Nieto 2024). Future studies should therefore use microbiome‐aware and metabolome‐aware designs without applying omics indiscriminately to every botanical intervention. The endpoint should match the hypothesized mechanism: Vinegar soaks should be evaluated for pH, irritation, and microbial change; fermented oral extracts for gut metabolites, stool microbiota, and systemic cytokines; lipid‐rich topicals for stratum‐corneum lipid composition, hydration, and TEWL; and antimicrobial botanicals for *S. aureus* adhesion, quorum sensing, virulence, biofilm formation, or bacterial‐product‐induced inflammation. Microbiome claims should also distinguish antimicrobial killing from ecological restoration because reducing *S. aureus* in vitro does not necessarily restore commensal diversity and broad antimicrobial activity may theoretically disrupt protective skin microbes. A more sophisticated botanical strategy would aim to attenuate *S. aureus* virulence or inflammation while preserving microbial diversity, thereby aligning botanical AD research with the current view of flares as ecological and immunological disturbances rather than as simple infections.

## 9 Delivery Technologies: Making Botanical Pharmacology Reproducible

Many plant‐derived compounds have poor aqueous solubility, chemical instability, low oral bioavailability, uncertain skin penetration, and variable local residence time. These limitations are not minor formulation details; they are core barriers to clinical translation (Kaushal and Awasthi 2025; Gowda 2024). A compound that suppresses NF‐κB in vitro in a stimulated cell system may fail clinically because it oxidizes, precipitates, remains in the stratum corneum, is washed off, or causes irritation at the concentration needed for viable epidermal exposure. Delivery science therefore represents a central component of botanical AD therapeutics, particularly for products intended to act locally on keratinocytes, mast cells, sensory nerves, immune infiltrates, or barrier lipids. Liposomes, microsponges, microneedles, polymeric systems, and carbon nanodots illustrate emerging solutions. Glabridin liposomes improved solubility and enhanced the antipruritic/barrier‐restorative profile of glabridin in a histamine‐induced AD model (Lu, Cheng, et al. 2024). Dissolvable microneedle patches increased the therapeutic effect of Jawoongo by improving transdermal delivery, reducing skin thickness and lowering IL‐4, IL‐6, IL‐13, iNOS, and TNF‐α more effectively than ointment alone (Jang et al. 2023). Naringenin microsponges provide another example of rational topical design, in which a plant‐derived flavonoid was formulated and optimized for AD‐directed delivery rather than applied as an unmodified compound (Nagula and Wairkar 2020). Advanced delivery reviews emphasize polymeric conjugates, nanocarriers, and transdermal systems as approaches for overcoming solubility limitations, improving local accumulation, and prolonging cutaneous residence time (Li, Xu, et al. 2024; Vyas et al. 2026). These technologies may also improve safety by lowering the required dose, reducing systemic exposure, and concentrating activity at the diseased site. However, they introduce formulation‐specific risks that must be evaluated directly: Microneedles can disrupt barrier integrity, nanoparticles require biocompatibility assessment, penetration enhancers may increase sensitizer exposure, and occlusive vehicles may worsen folliculitis or infection risk. These concerns are amplified in pediatric AD, where impaired skin, facial involvement, excoriation, high surface‐area‐to‐body‐weight ratio, and caregiver‐controlled application can substantially alter exposure.

**FIGURE 2:**
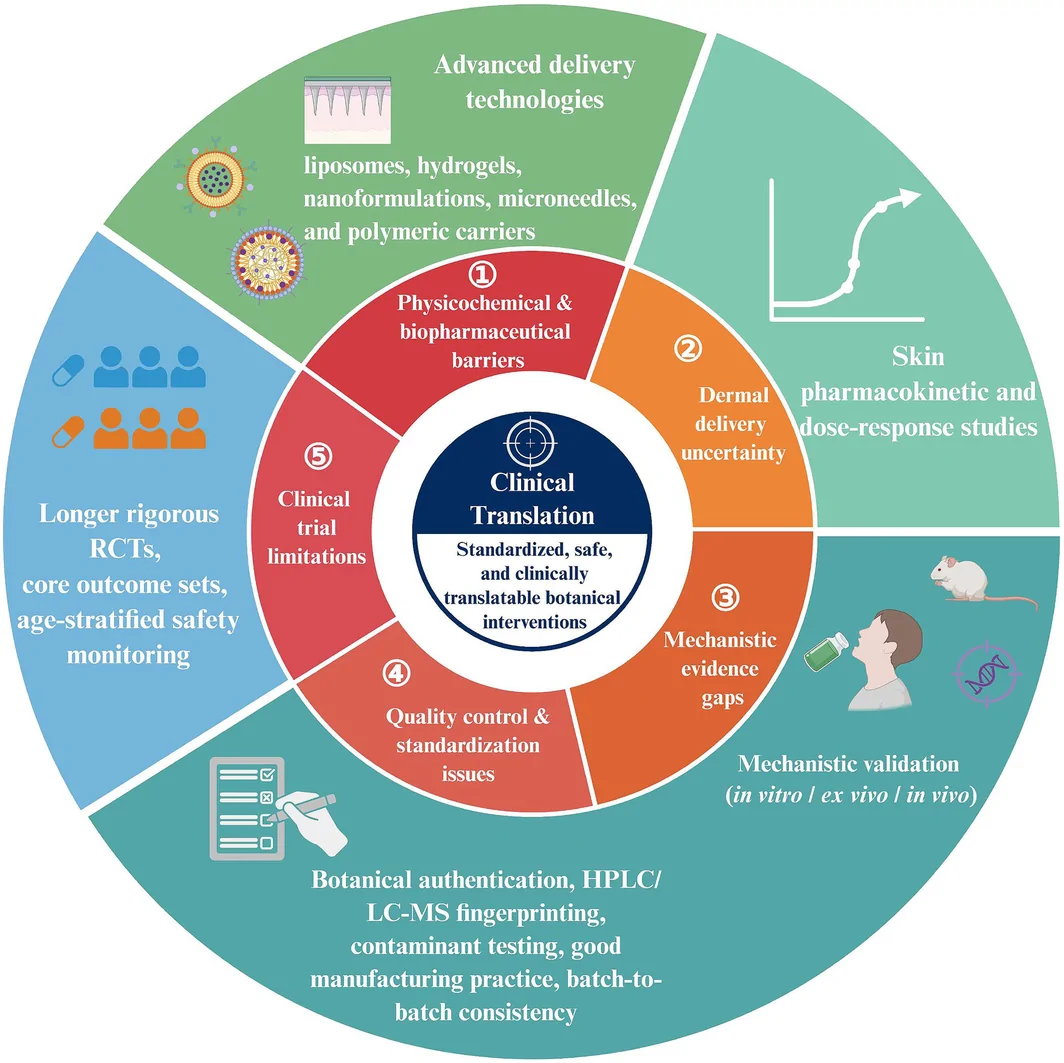
Current limitations of botanical interventions in atopic dermatitis and corresponding solutions. Physicochemical and biopharmaceutical barriers, dermal delivery uncertainty, mechanistic evidence gaps, quality‐control issues, and clinical‐trial limitations constrain the translation of botanical interventions for atopic dermatitis. Corresponding strategies include advanced delivery technologies, skin pharmacokinetic and dose–response studies, mechanistic validation, botanical authentication, HPLC/LC–MS fingerprinting, contaminant testing, good manufacturing practice, batch‐to‐batch consistency, longer rigorous randomized controlled trials, core outcome sets, and age‐stratified safety monitoring.

Reproducible delivery begins with reproducible material. Before liposomes, microneedles or nanocarriers are optimized, the botanical source must be authenticated, the extract chemically fingerprinted, marker compounds quantified, contaminants excluded, and batch‐to‐batch variation controlled. Otherwise, advanced delivery systems may simply transport an undefined and variable mixture more efficiently. Studies integrating chemical quality control with biological testing, such as JF decoction with HPLC/GC analysis and formula‐based metabolomic evaluation, provide a useful model for connecting phytochemical definition to pharmacodynamic interpretation (Li, Liang, et al. 2024). A rational translational pathway should begin with a target product profile: pediatric or adult use, topical or oral route, flare treatment or maintenance care, itch relief or barrier repair, monotherapy or adjunctive therapy. The formulation strategy should then be selected according to that profile, followed by definition of chemical markers and acceptable variation, vehicle or delivery‐platform optimization, skin penetration and irritation testing, pharmacodynamic biomarker evaluation in human‐relevant skin models and, finally, randomized clinical trials. This sequence is more demanding than repeating acute AD‐like mouse models with crude extracts, but it is essential if botanical products are to move from experimental plausibility to clinically credible, reproducible, and safe AD interventions. These translational barriers and corresponding solution strategies are summarized in Figure 2.

## 10 Toxicology, Pediatric Safety, and Product Quality

Safety is decisive in pediatric botanical AD care and must be weighed with efficacy rather than confined to a closing disclaimer. A product can be anti‐inflammatory in mice and still be inappropriate for children if it contains allergens, irritants, phototoxic constituents, heavy metals, pesticide residues, adulterants, microbial contamination, or variable active concentrations. Reviews of active naturals have already emphasized hepatotoxicity concerns for some oral Chinese herbal products and the need for caution despite perceived natural safety (Silverberg 2017). Adverse‐event reviews of emollient use show that even ostensibly benign topical products can produce harm, including irritation, allergic reactions, folliculitis, infection‐related issues, and other complications (Bhanot et al. 2019). Homemade walnut cream causing complicated infant AD is a concrete warning against unsupervised food‐based topical therapy (Abtahi‐Naeini et al. 2023). Allergic contact dermatitis is especially relevant in AD because barrier impairment and frequent topical exposure create favorable conditions for sensitization. Phloretin contact dermatitis in a patient with AD illustrates that luxury or botanical cosmetic ingredients are not exempt from allergenicity (Gatica‐Ortega and Pastor‐Nieto 2024). The pediatric spice and herb allergy literature further indicates that herb‐related reactions may be underdiagnosed and can range from mild oral symptoms to anaphylaxis (Berghea et al. 2025). For AD, this risk is not limited to ingestion; topical products made from nut oils, botanical fragrances, essential oils, or protein‐containing extracts can sensitize through inflamed skin.

Systemic botanical products require additional scrutiny because constituents may inhibit or induce cytochrome P450 (CYP) enzymes and P‐glycoprotein (P‐gp), alter coagulation or sedation, or add hepatic, renal, and immunologic toxicity. The strongest AD‐relevant examples concern cyclosporine: Grapefruit can increase exposure through intestinal CYP3A/P‐gp effects, whereas St John's wort can markedly reduce concentrations through induction, creating a clinically important loss‐of‐efficacy risk (Brunner et al. 1998; Bauer et al. 2003). Upadacitinib exposure is also affected by strong CYP3A inhibition or broad induction, so concentrated grapefruit products and St John's wort merit caution even though direct trials with specific AD botanicals are unavailable (Mohamed et al. 2017). These are not class effects of all plant foods; risk depends on constituent, dose, preparation, and chronicity. Dupilumab requires a different interpretation. As a monoclonal antibody, it is not a conventional CYP or P‐gp substrate, and a clinical CYP‐probe study found no clinically meaningful disease‐mediated change in CYP substrate exposure during dupilumab treatment (Davis et al. 2018). Direct pharmacokinetic interaction with most phytochemicals is therefore less plausible than with cyclosporine or upadacitinib. Nevertheless, evidence for co‐administered botanical products is sparse, and pharmacodynamic concerns remain: hypersensitivity, infection, overlapping immune effects, inconsistent adherence, or substitution of an unproven product for prescribed therapy. Compatibility studies should evaluate not only drug concentrations but also AD control, rescue medication, infections, liver/renal tests, eosinophilia, and product‐related allergy.

Pediatric adverse‐event reporting must be substantially more granular if botanical products are to be evaluated responsibly. Reports should specify age, weight, AD severity, body surface area treated, lesion location, skin integrity, amount applied, occlusion, bathing practices, concomitant medications, prior allergies, and timing of adverse events. Mild stinging or transient erythema should be distinguished from allergic contact dermatitis, infection, systemic symptoms, and laboratory abnormalities. Trials should actively assess sleep changes, sedation, gastrointestinal symptoms, and flare rebound after discontinuation; in infants, caregiver misuse and accidental ingestion should also be considered. Without such detail, claims of “no adverse events” provide limited clinical information. Risk communication should be explicit without being dismissive. Clinicians should not simply warn families that all herbal products are dangerous because this may encourage undisclosed use. A more constructive approach is to ask specifically about botanical products, examine ingredient lists, identify high‐risk components such as nut oils, fragrances and essential oils, discourage homemade preparations on inflamed skin, and recommend discontinuation when unexplained worsening occurs. This shared‐decision strategy respects patient preferences while maintaining a clear therapeutic hierarchy: Botanical products may have adjunctive value, but pediatric use requires defined composition, contaminant control, interaction awareness, site‐specific tolerability assessment, and active safety monitoring.

## 11 Research Gaps and Future Agenda

Future research should move from broad plant names to fully specified products. At minimum, reports should include Latin binomial and plant part; voucher specimen and authentication method; collection site, cultivar, harvest season, and storage; extraction solvent concentration, drug‐to‐solvent ratio, temperature, duration, cycles and yield; fermentation strain, inoculum, time, temperature, pH, and oxygen conditions; drying method; chromatographic fingerprint and quantitative marker ranges; contaminant and adulterant testing; stability; and batch identifiers. The ConPhyMP recommendations and the CONSORT extension for herbal interventions provide practical reporting frameworks (Gagnier et al. 2006; Heinrich et al. 2022).

Animal‐model selection also requires explicit limits. Repeated DNCB/DNFB challenge reproduces contact sensitization, epidermal injury, scratching, elevated IgE, and selected Th2/barrier features, but it does not reproduce the full genetic architecture, spontaneous onset, age‐dependent endotypes, environmental mixtures, chronic relapse‐remission course, systemic comorbidity, or microbiome ecology of human AD. Outcomes such as reduced ear thickness can reflect nonspecific anti‐irritant activity. Even refined DNCB protocols remain experimentally induced phenotypes (Riedl et al. 2023). Stronger evidence should triangulate across irritant‐sensitization, MC903, house‐dust‐mite and spontaneous/genetic models; include treatment withdrawal and rechallenge; and validate priority findings in reconstructed epidermis, human skin explants, or patient‐derived samples. No single model is expected to reproduce all AD dimensions. A practical research agenda can be organized into three tiers. Tier 1 studies should be small pharmacodynamic trials in adults or older adolescents using chemically standardized products and mechanism‐matched biomarkers. Tier 2 studies should be randomized vehicle‐ or placebo‐controlled trials in defined mild‐to‐moderate AD populations, incorporating validated severity, itch, sleep, barrier, and safety endpoints. Tier 3 studies should be pragmatic adjunctive trials within standard care, testing whether botanical products reduce flares, improve adherence, lower rescue medication use, or reduce caregiver burden without increasing adverse events. Such studies should also consider integration with biologics and JAK inhibitors, where botanicals may be most useful as adjuncts for barrier comfort, residual itch, maintenance care, or corticosteroid‐sparing support. Data transparency is essential: Protocols, chemical fingerprints, chromatograms, adverse‐event tables and rescue‐medication algorithms should be publicly available before botanical products can be considered reproducible and clinically interpretable.

## 12 Conclusion

This review's specific contribution is to integrate food‐source provenance, functional‐food and nutraceutical concepts, processing‐dependent chemistry, food‐matrix and oral‐bioavailability constraints, gut‐skin evidence, pediatric safety, herb–drug compatibility, delivery science and evidence strength within one AD framework. Food‐derived flavonoids, polysaccharides, saponins, oils, and fermented products may support barrier, itch, or inflammatory control, but edible origin and pathway modulation do not directly establish therapeutic benefit. Human evidence includes encouraging, neutral, and cautionary findings; preclinical metabolomics and DNCB/DNFB studies remain hypothesis‐generating unless supported by functional and human‐relevant validation. The most defensible role for a standardized botanical product is therefore adjunctive: Improving a specified residual outcome while guideline‐based anti‐inflammatory care remains intact. Progress requires reproducible processing and chemical specifications, measured exposure, comparator‐controlled trials, long‐term and pediatric safety surveillance, and transparent interaction studies with cyclosporine, biologics, and JAK inhibitors.

## Author Contributions

**Qianlan Yuan:** investigation, visualization. **Muhuang Zeng:** conceptualization, investigation, validation, methodology, writing – review and editing. **Xiaofei Lu:** conceptualization, methodology, writing – review and editing. **Shuojia Huang:** investigation, visualization.

## Funding

The authors have nothing to report.

## Ethics Statement

All animal procedures were conducted in accordance with the ARRIVE (Animal Research Reporting of In Vivo Experiment) guidelines for the care and use of laboratory animals.

## Conflicts of Interest

The authors declare no conflicts of interest.

## Data Availability

The data that support the findings of this study are available from the corresponding author upon reasonable request.
